# Association of HbA1c and comorbidities on stroke severity: insights from a cross-sectional analysis in a tertiary hospital

**DOI:** 10.3389/fneur.2026.1721769

**Published:** 2026-04-30

**Authors:** Bader N. Alharbi, Fahad K. Alsharef, Fahad K. Aldossary, Hamad A. Alsaggabi, Saleh A. Alhawery, Hussain S. Aldera, Moeber M. Mahzari, Nasser Alotaibi, Bijesh Yadav, Naif M. Alhawiti

**Affiliations:** 1 Department of Basic Medical Sciences, College of Medicine at King Saud bin Abdulaziz University for Health Sciences, Riyadh, Saudi Arabia; 2 King Abdullah International Medical Research Centre, Ministry of the National Guard Health Affairs, Riyadh, Saudi Arabia; 3 College of Medicine, King Saud bin Abdulaziz University for Health Sciences, Riyadh, Saudi Arabia; 4 Department of Medicine, King Abdulaziz Medical City, Riyadh, Saudi Arabia; 5 Division of Neurology, Department of Medicine, King Abdulaziz Medical City, National Guard Health Affairs, Riyadh, Saudi Arabia; 6 Department of Clinical Laboratory Sciences, College of Applied Medical Sciences, King Saud bin Abdulaziz University for Health Sciences, Riyadh, Saudi Arabia

**Keywords:** comorbidities, diabetes mellitus, HbA1c, NIHSS, stroke severity

## Abstract

**Introduction:**

Elevated HbA1c levels, in combination with comorbidities such as diabetes and hypertension, have been linked to increased stroke severity, poorer functional recovery, and higher mortality. However, the predictive value of HbA1c—a marker of long-term glycemic control—for acute stroke severity remains unclear. This study investigated the relationship between admission HbA1c levels and comorbidities on stroke severity in patients presenting to a tertiary care center.

**Methods:**

We conducted a retrospective cross-sectional study at King Abdulaziz Medical City in Riyadh, Saudi Arabia, including 672 adult patients with acute ischemic stroke between January 2016 and January 2023. Patients were stratified into three groups based on admission HbA1c (≤6.4%, 6.5–7.9%, ≥8%). Stroke severity was assessed using the National Institutes of Health Stroke Scale (NIHSS) at admission and discharge.

**Results:**

A total of 672 patients were included (mean age 63.7 ± 13.2 years; 68.6% male). At admission, 49.9% had mild, 42.3% moderate, and 7.9% severe strokes, with no significant association between HbA1c and stroke severity (*p* = 0.177). Although higher HbA1c levels were associated with vascular comorbidities in bivariate analysis, only LDL (OR = 1.06, 95% CI: 1.00–1.12; *p* = 0.045) and atrial fibrillation (OR = 0.73, 95% CI: 0.58–0.91; *p* = 0.005) remained significant after adjustment. Antihypertensive use was independently associated with higher HbA1c (OR = 1.53, 95% CI: 1.30–1.80; *p* < 0.001), while NOAC use showed an inverse association (OR = 0.74, 95% CI: 0.55–0.99; *p* = 0.040). In multivariable analysis, atrial fibrillation was the only independent predictor of increased stroke severity, whereas HbA1c and other vascular risk factors were not significant. Stroke severity strongly predicted in-hospital mortality (OR = 1.27, 95% CI: 1.02–1.59; *p* = 0.032), prolonged hospitalization (OR = 1.31, 95% CI: 1.15–1.49; *p* < 0.001), and stroke-related death (OR = 2.18, 95% CI: 1.37–3.47; *p* = 0.002), while HbA1c was not associated with these outcomes.

**Discussion:**

In conclusion, HbA1c was not associated with acute stroke severity. While it remains a valuable marker of long-term vascular risk, its role in predicting acute neurological injury appears limited. In contrast, established clinical tools—particularly the NIHSS—remain the most reliable instruments for early prognostic evaluation of stroke severity.

## 1 Introduction

Ischemic stroke remains a major global cause of mortality and long-term disability, with diabetes mellitus (DM) recognized as a significant contributor to this burden (1–3). Patients with coexisting comorbidities face nearly double the risk of ischemic stroke and exhibit approximately 20% higher mortality compared to non-diabetic individuals (4). Moreover, they frequently present with more severe neurological deficits and experience poorer recovery following stroke (5, 6, 35). The coexistence of additional comorbidities, such as hypertension, hyperlipidemia, coronary artery disease, and atrial fibrillation, further exacerbates stroke severity, hinders functional recovery, and increases mortality rates (4, 7). Acute hyperglycemia, observed in 20–40% of stroke patients, has also been consistently linked to larger infarct volumes, higher stroke severity scores, and unfavorable clinical outcomes (6–8). However, much of the existing research has emphasized admission glucose levels, which are prone to fluctuation under acute stress, rather than chronic glycemic control. Glycated hemoglobin (HbA1c), which reflects average plasma glucose levels over the preceding 8–12 weeks, offers a more reliable marker of long-term glycemic regulation (9). Unlike single glucose measurements, HbA1c is less influenced by acute stress and may therefore provide greater prognostic value in ischemic stroke.

Although HbA1c has potential as a prognostic marker, evidence regarding its association with acute stroke severity remains inconsistent. Certain studies suggest that elevated HbA1c is linked to more severe neurological deficits and poorer functional outcomes, particularly in patients with comorbidities such as hypertension and DM (10, 11). Conversely, other investigations using standardized severity assessments, such as the National Institutes of Health Stroke Scale (NIHSS), have reported no significant association (12, 13). These discrepancies may be attributed to variations in HbA1c thresholds, outcome definitions, and study populations. Clarifying this relationship holds important clinical implications, as HbA1c is a modifiable parameter and a routinely available laboratory test. Establishing its prognostic utility in the context of acute ischemic stroke could strengthen risk stratification, guide early therapeutic decision-making, and enhance patient counseling regarding long-term vascular health.

While HbA1c is an established marker of long-term vascular risk and has been linked to post-stroke functional outcomes, its role in predicting acute neurological severity in the presence of comorbid conditions remains uncertain. To address this gap, the present study sought to examine the relationship between admission HbA1c levels and coexisting comorbidities on stroke severity as defined by the NIHSS in a large cohort of patients admitted to a tertiary care hospital.

## 2 Methods

### 2.1 Study design and setting

This retrospective cross-sectional study was carried out in the Neurology Division of King Abdulaziz Medical City (KAMC), a tertiary care facility in Riyadh, Saudi Arabia. This medical city poses an electronic health record system called (BESTCare) in which medical professionals ensure all relevant clinical data are entered as much as possible. The patients labeled by ER with diagnosis of stroke are admitted in a specialized stroke unit. After admission, history, examination, laboratory, and radiological findings are obtained and recorded by a neurologist and entered in the system. KAIMRC, King Abdullah International Medical Research Center (the supervising institutional board review), granted the study group access to BESTCare system. The clinical data of a total of 2,646 patients admitted to this stroke unit with diagnosis of stroke between January 1, 2016, to January 1, 2023, were retrieved. All those patients were screened and considered as potential candidates. After application of the predefined inclusion criteria a total of 672 patients were found eligible.

### 2.2 Participants

The study included adults aged 18 years and older with a confirmed diagnosis of ischemic stroke. Patients were excluded if they were pregnant, had experienced a transient ischemic attack (TIA), or had a hemorrhagic stroke, to maintain focus on the ischemic subtype, which has a well-established link with glycemic status (14). A total of 672 patients met these criteria and were included in the final analysis. Data were retrospectively extracted and recorded in Microsoft Excel using a standardized, IRB-approved collection form. All datasets were securely stored within the National Guard Health Affairs (NGHA) network on a password-protected computer maintained by the principal investigator. Based on the American Diabetes Association (ADA) guidelines, patients were categorized into three groups according to admission HbA1c levels: non-diabetic/pre-diabetic (≤ 6.4%), controlled diabetes (6.5–7.9%), and poorly controlled diabetes (≥8%) (15).

### 2.3 Sample size estimation

The sample size was estimated using OpenEpi (Version 3) based on standard epidemiological assumptions. Due to the absence of precise national prevalence data for ischemic stroke in Saudi Arabia, an estimated incidence of 2% reported in prior population-based studies was used as a reference. Using a 95% confidence level, 5% margin of error, and a finite population size of 5,000, the minimum required sample size was calculated as 357 participants.

To enhance statistical power and minimize selection bias, all eligible patients identified during the study period were included, resulting in a final cohort of 672 patients, which exceeds the minimum requirement.

### 2.4 Variables and outcome measures

The primary outcome of this study was stroke severity, evaluated using the NIHSS at both admission and discharge. Stroke severity was assessed using the National Institutes of Health Stroke Scale (NIHSS), a standardized and validated tool for quantifying neurological impairment after stroke (16). Given that NIHSS is a continuous scale without predefined categorical thresholds, patients were stratified into mild (0–4), moderate (5–15), and severe (≥16) groups based on commonly used groupings in the literature (17), with ≥16 cutoff is supported by its established prognostic significance (18).

Secondary variables encompassed demographic characteristics (age and sex); comorbid conditions including hypertension, DM, hyperlipidemia, coronary artery disease (CAD), atrial fibrillation (AF), valvular heart disease, prior stroke, and TIA; and smoking status (smoker or non-smoker). Comorbid conditions were identified based on documented physician diagnosis recorded in the BESTCare electronic medical records.

Laboratory parameters included low-density lipoprotein (LDL) and high-density lipoprotein (HDL). Hyperlipidemia was defined as total cholesterol ≥240 mg/dL and/or LDL ≥ 100 mg/dL, while low HDL was defined as <60 mg/dL, in accordance with institutional laboratory standards (19). HbA1c levels were measured in the hospital’s central laboratory using high-performance liquid chromatography (HPLC) following standard NGSP/IFCC calibration procedures.

Medication history included the use of antiplatelet agents (aspirin, ticagrelor, clopidogrel), anticoagulants (non-vitamin K oral anticoagulants [NOACs] and warfarin), antihypertensive agents, and statins. During our review of the available clinical data, anti-hyperglycemic medication administration prior to admission was not recorded in the vast majority of patients, therefore, it was not possible to be analyzed.

Additional outcomes assessed were in-hospital mortality, cause of death, and length of hospital stay (LOS). Body mass index (BMI) was categorized according to standard thresholds: underweight (<18.5 kg/m^2^), normal (18.5–24.9 kg/m^2^), overweight (25–29.9 kg/m^2^), and obese (≥30 kg/m^2^) (20).

### 2.5 Statistical analysis

All statistical analyses were conducted using IBM SPSS Statistics version 25.0 (IBM Corp., Armonk, NY, USA). Categorical variables were summarized as frequencies and percentages, while continuous variables were reported as means ± standard deviations. Associations between HbA1c categories and NIHSS Classification and all the other categorical variables were assessed using Pearson’s chi-square test; when test assumptions were not met, the likelihood ratio or linear-by-linear association tests were applied. Differences in continuous variables across HbA1c groups were evaluated using one-way analysis of variance (ANOVA). A *p*-value < 0.05 was considered statistically significant.

To identify independent associations, multivariable analyses were performed. Multinomial logistic regression was used to evaluate factors associated with HbA1c categories and to assess predictors of NIHSS scores at admission and related outcomes. Results from regression analyses were reported as OR with corresponding 95% confidence intervals to aid interpretability. A *p*-value < 0.05 was considered statistically significant for all analyses.

Missing data were minimal (<5% for all variables) and were not imputed. Analyses were conducted on available cases, and the small extent of missingness was considered unlikely to bias results or affect validity. NIHSS scores at discharge were available for 59.2% of patients and analyzed as a secondary outcome. Since admission NIHSS, the primary outcome, was complete for all patients, the partial discharge data were not expected to compromise the robustness of the main analyses.

### 2.6 Ethical considerations

Ethical approval for this study was granted by the Institutional Review Board of King Abdullah International Medical Research Center (KAIMRC, Protocol No. SP23R/107/06). Given the retrospective design and use of de-identified data, the requirement for informed consent was waived. All data were managed in accordance with institutional guidelines to maintain confidentiality and ensure data security.

## 3 Results

### 3.1 Study population characteristics

A total of 672 patients with acute ischemic stroke were included (mean age 63.7 ± 13.2 years; 68.6% male). Obesity (BMI ≥ 30 kg/m^2^) was present in 39.1%. The most frequent comorbidities were hypertension (74.0%), diabetes mellitus (65.2%), and hyperlipidemia (36.5%). Coronary artery disease was noted in 13.5%, and 17.7% were current or former smokers. Laboratory data showed elevated LDL (≥100 mg/dL) in 58.8% and low HDL (<60 mg/dL) in 97.6% (Table 1).

**Table 1:** Baseline demographic and clinical characteristics of the study population.

Categories	Characteristic	Total (*n* = 672)
Age (year)	Age, years (mean ± SD)	63.7 ± 13.2
Gender (male/female)	Male, *n* (%)	461 (68.6)
	Female, *n* (%)	211 (31.4)
Anthropometrics (%)	BMI ≥ 30 kg/m^2^ (Obese), *n* (%)	262 (39.1)
Vascular risk factors	Hypertension, *n* (%)	497 (74.0)
	Diabetes mellitus, *n* (%)	438 (65.2)
	Hyperlipidemia, *n* (%)	245 (36.5)
	Coronary artery disease, *n* (%)	91 (13.5)
	Current or former smokers, *n* (%)	119 (17.7)
Laboratory values	HDL < 60 mg/dL, *n* (%)	656 (97.6)
	LDL ≥ 100 mg/dL, *n* (%)	395 (58.8)

### 3.2 Impact of admission HbA1c levels on stroke severity

**Figure 1:**
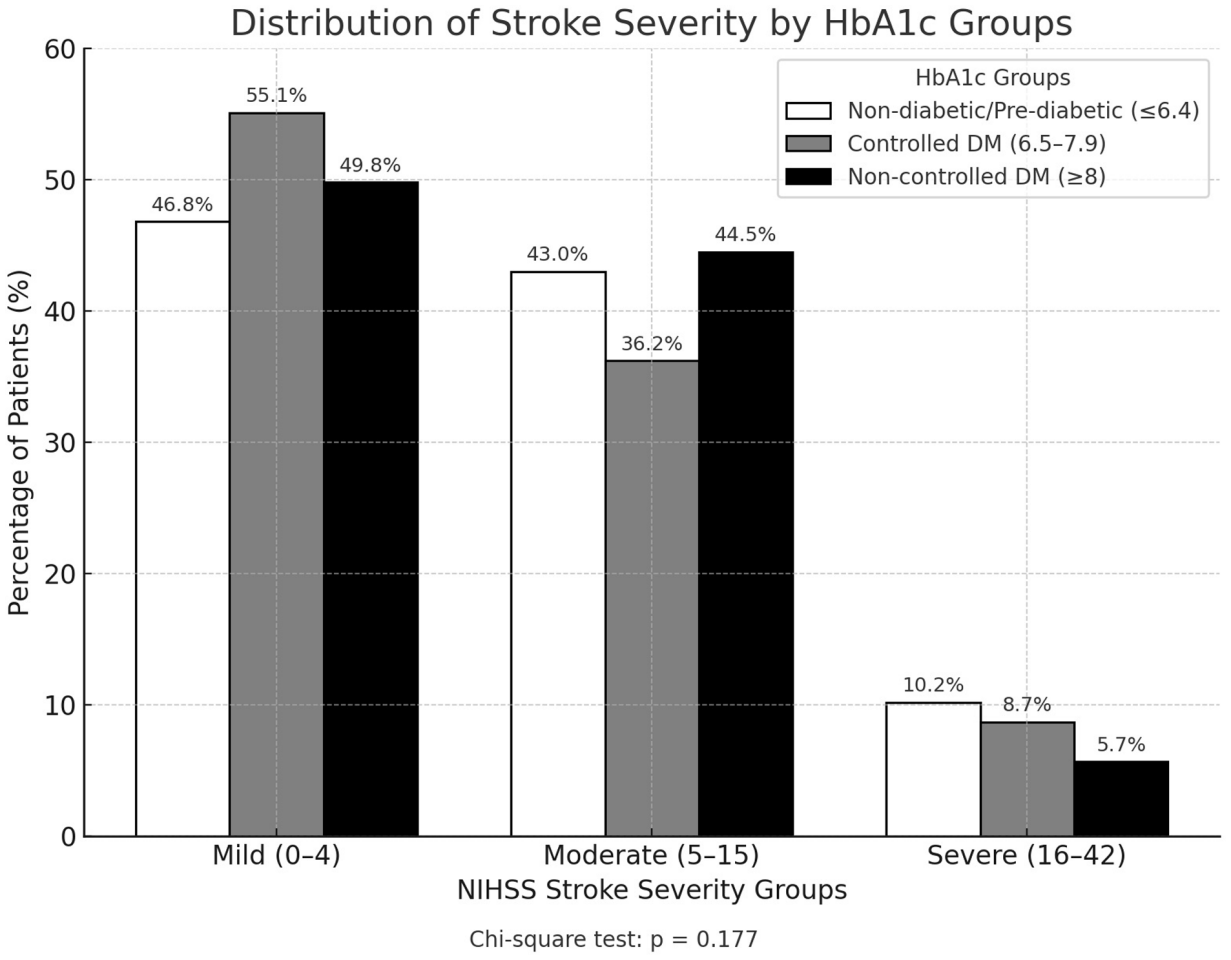
Stroke severity at admission stratified by HbA1c groups.

At admission, nearly half of patients presented with mild stroke (49.9%), 42.3% with moderate, and 7.9% with severe strokes. Stroke severity distribution did not differ significantly across HbA1c groups (*p* = 0.177) (Table 2; Figure 1).

**Table 2:** Distribution of stroke severity across HbA1c categories.

NIHSS categories	Non-diabetic/Pre-diabetic (≤6.4%)	Controlled DM (6.5–7.9%)	Poorly controlled DM (≥8%)	Total (n = 672)	*p*-value
Mild (0–4)	110 (46.8%)	76 (55.1%)	149 (49.8%)	335 (49.9%)	
Moderate (5–15)	101 (43.0%)	50 (36.2%)	133 (44.5%)	284 (42.3%)	
Severe (16–42)	24 (10.2%)	12 (8.7%)	17 (5.7%)	53 (7.9%)	
Overall comparison (χ^2^ test)					0.177

At discharge, NIHSS data were available for 398 patients. Most had mild strokes (70.9%), 25.9% were moderate, and 3.3% severe. Similar to admission, stroke severity was not significantly associated with HbA1c (*p* = 0.682).

### 3.3 Multivariable analysis of factors associated with stroke severity

In the multivariable ordinal logistic regression model (Table 3), HbA1c levels were not independently associated with stroke severity. Compared with patients with HbA1c ≥ 8%, those with HbA1c ≤ 6.4% and 6.5–7.9% did not demonstrate a statistically significant difference in stroke severity after adjustment.

**Table 3:** Multivariable ordinal logistic regression model of HbA1c and clinical factors associated with stroke severity.

Variable	Adjusted OR	95% CI	*p*-value
Age (per year)	1.02	1.00–1.03	0.028
HbA1c (Ref: ≥8%)			
≤6.4%	1.44	0.88–2.37	0.148
6.5–7.9%	0.88	0.58–1.33	0.546
Sex (male vs. female)	1.34	0.95–1.88	0.097
Hypertension (yes vs. no)	0.97	0.66–1.51	0.873
Diabetes mellitus (yes vs. no)	0.72	0.44–1.18	0.178
Hyperlipidemia (yes vs. no)	1.23	0.89–1.71	0.203
Coronary artery disease (yes vs. no)	0.92	0.59–1.43	0.701
Smoking (yes vs. no)	1.41	0.93–2.17	0.108
Atrial fibrillation (yes vs. no)	**2.87**	**1.64–5.03**	**<0.001**

Bold values indicate statistically significant results (*p* < 0.05).

Among the evaluated risk factors, atrial fibrillation emerged as the strongest independent predictor of increased stroke severity, demonstrating a significant association with higher NIHSS categories. Increasing age was also independently associated with greater stroke severity, although the magnitude of effect was modest.

In contrast, traditional vascular risk factors—including hypertension, diabetes mellitus, hyperlipidemia, coronary artery disease, and smoking—were not independently associated with stroke severity after multivariable adjustment (Table 3).

### 3.4 Association of HbA1c levels with comorbid conditions

Patients with higher HbA1c were more likely to have hypertension (85.5% controlled DM, 81.6% poorly controlled vs. 57.4% non-diabetic; *p* < 0.001), hyperlipidemia (43.5 and 42.1% vs. 25.1%; *p* < 0.001), and coronary artery disease (21.7 and 14.4% vs. 7.7%; *p* = 0.001) in bivariate analysis. However, after adjustment for potential confounding variables in multivariable analysis, these associations were no longer statistically significant.

Among the variables analyzed, only LDL levels and atrial fibrillation retained statistical significance in the multivariable model. Elevated LDL levels were independently associated with higher HbA1c categories (OR = 1.06, 95% CI: 1.00–1.12; *p* = 0.045), while atrial fibrillation showed an inverse association with HbA1c levels (OR = 0.73, 95% CI: 0.58–0.91; *p* = 0.005). Diabetes mellitus remained strongly associated with HbA1c levels (OR = 3.82, 95% CI: 3.33–4.39; *p* < 0.001) (Table 4).

**Table 4:** Association of HbA1c categories with comorbid conditions.

Variables	Non-diabetic/Pre-diabetic (*n* = 235) %	Controlled DM (*n* = 138) %	Poorly controlled DM (*n* = 299) %	*p*-value (Bivariate)	OR (95% CI)	*p*-value (Multivariate)
BMI group	<18.5: 10 (4.3) 18.5–24.9: 55 (23.4) 25–29.9: 80 (34.0) ≥30: 90 (38.3)	<18.5: 3 (2.2) 18.5–24.9: 32 (23.2) 25–29.9: 44 (31.9) ≥30: 59 (42.8)	<18.5: 5 (1.7) 18.5–24.9: 56 (18.9) 25–29.9: 123 (41.4) ≥30: 113 (38.0)	0.194	0.99 (0.98–1.01)	0.316
Elevated LDL levels (≥100 mg/dL)	137 (58.3)	79 (57.2)	179 (59.9)	0.860	1.06 (1.00–1.12)	0.045
Suboptimal HDL levels (<60 mg/dL)	229 (97.4)	138 (100.0)	289 (96.7)	0.101	0.92 (0.70–1.20)	0.524
Smoking	50 (21.3)	28 (20.3)	41 (13.7)	0.051	1.00 (0.84–1.19)	0.978
Hypertension	135 (57.4)	118 (85.5)	244 (81.6)	<0.001	0.97 (0.83–1.13)	0.663
Diabetes Mellitus	43 (18.3)	107 (77.5)	288 (96.3)	<0.001	3.82 (3.33–4.39)	<0.001
Hyperlipidemia	59 (25.1)	60 (43.5)	126 (42.1)	<0.001	1.04 (0.91–1.18)	0.604
Coronary Artery Disease	18 (7.7)	30 (21.7)	43 (14.4)	0.001	1.00 (0.84–1.19)	0.979
Atrial Fibrillation	27 (11.5)	13 (9.4)	16 (5.4)	0.034	0.73 (0.58–0.91)	0.005

### 3.5 Association of HbA1c levels with medication use

Medication use varied by HbA1c category. Statin therapy was most frequent among patients with poorly controlled diabetes (91.3%) compared to non-diabetic/pre-diabetic patients (84.7%) (*p* = 0.024) in bivariate analysis; however, this association did not remain statistically significant after multivariable adjustment (OR = 1.20, 95% CI: 0.97–1.49; *p* = 0.090). Antihypertensive use was also higher in poorly controlled diabetes (86.3%) than in non-diabetic/pre-diabetic patients (67.2%) (*p* < 0.001), and this association remained significant in multivariable analysis (OR = 1.53, 95% CI: 1.30–1.80; *p* < 0.001).

Plavix use showed a significant difference across groups in unadjusted analysis (*p* = 0.004), but this did not persist after adjustment (OR = 1.10, 95% CI: 0.95–1.27; *p* = 0.200). Although NOAC use did not reach statistical significance in bivariate analysis (*p* = 0.070), it demonstrated a significant inverse association in the adjusted model (OR = 0.74, 95% CI: 0.55–0.99; *p* = 0.040) (Table 5).

**Table 5:** Association between HbA1c categories and medication use.

Drugs	Non-diabetic/Pre-diabetic (*n* = 235) %	Controlled DM (*n* = 138) %	Poorly controlled DM (*n* = 299) %	*p*-value (Bivariate)	OR (95% CI)	*p*-value (Multivariate)
Statin use	199 (84.7)	123 (89.13)	273 (91.3)	0.024	1.20 (0.97–1.49)	0.090
Plavix use	122 (51.9)	95 (68.8)	185 (61.9)	0.004	1.10 (0.95–1.27)	0.200
Aspirin use	186 (79.1)	113 (81.9)	244 (81.6)	0.725	0.95 (0.79–1.14)	0.588
NOACs use	22 (9.4)	7 (5.1)	14 (4.7)	0.070	**0.74 (0.55–0.99)**	**0.040**
Warfarin use	23 (9.8)	9 (6.5)	19 (6.4)	0.288	0.88 (0.68–1.15)	0.350
Anti-HTN use	158 (67.2)	118 (85.5)	258 (86.3)	<0.001	**1.53 (1.30–1.80)**	**<0.001**

Bold values indicate statistically significant results (*p* < 0.05).

### 3.6 Association of HbA1c levels with stroke outcomes and in-hospital course

Overall in-hospital mortality was 4.6% (31 patients), with no significant variation observed across HbA1c groups (*p* = 0.905), and this lack of association remained consistent after multivariable adjustment (OR = 0.94, 95% CI: 0.68–1.29; *p* = 0.686). Stroke-related mortality occurred in 1.3% of patients and similarly showed no statistically significant difference across HbA1c categories in both unadjusted (*p* = 0.140) and adjusted analyses (OR = 0.60, 95% CI: 0.29–1.23; *p* = 0.157). Prolonged hospital stay (≥25 days) was documented in 11.9% of cases but was not associated with HbA1c level in bivariate analysis (*p* = 0.715), and this finding remained non-significant after controlling for potential confounders (OR = 0.99, 95% CI: 0.95–1.03; *p* = 0.598) (Table 6).

**Table 6:** Stroke outcomes and hospital course by HbA1c categories.

Outcomes	Non-diabetic/Pre-diabetic (*n* = 235) %	Controlled DM (*n* = 138) %	Poorly controlled DM (*n* = 299) %	*p*-value (Bivariate)	OR (95% CI)	*p*-value (Multivariate)
In-hospital mortality	12 (5.1)	6 (4.3)	13 (4.3)	0.905	0.94 (0.68–1.29)	0.686
Stroke-related deaths	2 (0.9)	4 (2.9)	3 (1.0)	0.140	0.60 (0.29–1.23)	0.157
Length of stay ≥25 days	24 (10.3)	16 (11.6)	40 (13.4)	0.715	0.99 (0.95–1.03)	0.598

### 3.7 Admission stroke severity and clinical outcomes based on NIHSS

Stroke severity at admission showed a strong association with subsequent clinical outcomes. Patients with severe strokes had the highest in-hospital mortality (13.2% vs. 3.6% in mild and 4.2% in moderate cases; *p* = 0.007), which remained significant after adjustment (OR = 1.27, 95% CI: 1.02–1.59; *p* = 0.032). Prolonged hospitalization was also more frequent in severe cases (35.8% vs. 3.9% in mild; *p* < 0.001) and remained independently associated (OR = 1.31, 95% CI: 1.15–1.49; *p* < 0.001). Stroke-related mortality was markedly higher in severe strokes (9.4% vs. <1%; *p* < 0.001), with a persistent adjusted association (OR = 2.18, 95% CI: 1.37–3.47; *p* = 0.002). Comorbidities differed by stroke severity, with atrial fibrillation most frequent in severe cases (22.6% vs. 4.5% mild; *p* < 0.001), retaining significance after adjustment (OR = 1.34, 95% CI: 1.09–1.65; *p* = 0.006). Coronary artery disease was more common in severe strokes but was not significant after adjustment. Other variables showed no independent association. Treatment patterns varied, as patients with severe strokes were less likely to receive clopidogrel (*p* < 0.001), which remained significant (OR = 0.68, *p* < 0.001). NOAC use was not significant after adjustment, while antihypertensive use showed a modest association (OR = 1.20; *p* = 0.014) (Table 7).

**Table 7:** Clinical variables according to admission NIHSS severity categories.

Variables	Mild (*n* = 335) %	Moderate (*n* = 284) %	Severe (*n* = 53) %	*P*-value (Bivariate)	OR (95% CI)	*p*-value (Multivariate)
BMI (kg/m^2^) group	<18.5: 12 (3.6) 18.5–24.9: 65 (19.4) 25–29.9: 130 (38.8) ≥30: 128 (38.2)	<18.5: 5 (1.8) 18.5–24.9: 63 (22) 25–29.9: 99 (35.1) ≥30: 115 (40.8)	<18.5: 1 (1.9) 18.5–24.9: 15 (28) 25–29.9: 18 (34.0) ≥30: 19 (35.8)	0.538	1.00 (0.99–1.01)	0.524
Smoking	58 (17.3)	51 (18.0)	10 (18.9)	0.953	0.020 (0.010–0.146)	0.758
Elevated LDL levels (≥100 mg/dL)	196 (58.5)	172 (60.6)	27 (50.9)	0.422	0.99 (0.94–1.03)	0.522
Suboptimal HDL levels (<60 mg/dL)	328 (97.9)	276 (97.2)	52 (98.1)	0.815	0.97 (0.80–1.18)	0.785
Hypertension	246 (73.4)	209 (73.6)	42 (79.2)	0.658	0.96 (0.83–1.10)	0.510
Diabetes mellitus	215 (64.2)	192 (67.6)	31 (58.5)	0.381	1.01 (0.91–1.12)	0.863
Coronary artery disease	46 (13.7)	32 (11.3)	13 (24.5)	0.035	1.03 (0.90–1.19)	0.644
Atrial fibrillation	15 (4.5)	29 (10.2)	12 (22.6)	<0.001	1.34 (1.09–1.65)	0.006
Hyperlipidemia	122 (36.4)	114 (40.1)	9 (17.0)	0.006	0.95 (0.86–1.06)	0.361
Plavix use	245 (73.1)	143 (50.4)	14 (26.4)	<0.001	0.68 (0.62–0.76)	<0.001
NOACs use	13 (3.9)	25 (8.8)	5 (9.4)	0.029	0.92 (0.73–1.15)	0.458
Aspirin use	276 (82.4)	230 (81.0)	37 (69.8)	0.096	1.05 (0.92–1.20)	0.449
Statin use	298 (89.0)	257 (90.5)	44 (83.0)	0.273	0.95 (0.82–1.11)	0.523
Anti-HTN use	254 (75.8)	236 (83.1)	44 (83.0)	0.066	1.20 (1.04–1.38)	0.014
Warfarin use	21 (6.3)	24 (8.5)	6 (11.3)	0.335	0.91 (0.76–1.11)	0.357
Mortality	12 (3.6)	12 (4.2)	7 (13.2)	0.007	1.27 (1.02–1.59)	0.032
Stroke-related deaths	2 (0.6)	2 (0.7)	5 (9.4)	<0.001	2.18 (1.37–3.47)	0.002
Length of stay ≥25 days	13 (3.9)	48 (16.9)	19 (35.8)	<0.001	1.31 (1.15–1.49)	<0.001

### 3.8 Stroke severity: admission to discharge

Stroke severity improved significantly during hospitalization (*p* < 0.001). Mild strokes increased from 49.9% at admission to 70.9% at discharge, while severe strokes decreased from 7.9 to 3.3% (Table 8).

**Table 8:** Change in stroke severity from admission to discharge.

NIHSS category	At admission (*n* = 672)	At discharge (*n* = 398)	*p*-value
Mild (0–4)	335 (49.9%)	282 (70.9%)	
Moderate (5–15)	284 (42.3%)	103 (25.9%)	
Severe (16–42)	53 (7.9%)	13 (3.3%)	
Overall comparison (χ^2^ test)			<0.001

## 4 Discussion

In this retrospective study of 672 patients with acute ischemic stroke, admission HbA1c levels showed no association with stroke severity, as measured by the NIHSS (Table 2). However, HbA1c categories were significantly associated with several vascular comorbidities, including hypertension, hyperlipidemia, coronary artery disease, and atrial fibrillation. These findings suggest that while HbA1c may not independently reflect acute neurological severity at presentation, it remains closely linked to the broader vascular risk profile of patients with ischemic stroke. Patients were categorized into three HbA1c groups: non-diabetic/pre-diabetic (≤ 6.4%, *n* = 235), controlled diabetes (6.5–7.9%, *n* = 138), and poorly controlled diabetes (≥8%, *n* = 299). The coexisting comorbid condition within stroke patients may potentiate the severity of stroke. As shown, hypertension was significantly more prevalent among diabetic patients, occurring in 85.5% of the controlled and 81.6% of the poorly controlled groups, compared with 57.4% of non-diabetic/pre-diabetic patients (*p* < 0.001). Likewise, hyperlipidemia was more frequent in controlled (43.5%) and poorly controlled diabetes (42.1%) than in the non-diabetic/pre-diabetic group (25.1%, *p* < 0.001). CAD prevalence was highest in controlled diabetes (21.7%), followed by poorly controlled diabetes (14.4%) and non-diabetic/pre-diabetic patients (7.7%, *p* = 0.001). According to Cipolla et al., comorbidities such as hypertension may elevate pulse pressure and shear stress on the endothelium, leading to blood–brain barrier disruption and vascular wall injury, thereby worsening stroke-related outcomes (21). Additionally, the literature identifies both hyperlipidemia and CAD as important vascular risk factors that aggravate stroke severity. Hyperlipidemia contributes to atherosclerotic plaque development and endothelial dysfunction, increasing the likelihood of large-vessel occlusion and more severe ischemic events. Likewise, CAD represents a systemic atherosclerotic burden and impaired cardiac function, which heighten the risk of cardioembolic events and reduced cerebral perfusion. Together, these mechanisms account for the observed link between hyperlipidemia, CAD, and greater stroke severity at admission (22–25). In our cohort, these comorbidities were more frequent across higher HbA1c categories, although HbA1c itself was not significantly associated with NIHSS-defined stroke severity at admission.

Patients with poorly controlled diabetes (HbA1c ≥ 8%) also showed higher use of statins and antihypertensive medications, which is consistent with a greater burden of vascular comorbidity. Clopidogrel use also differed significantly across HbA1c groups, whereas aspirin, NOACs, and warfarin did not. Stroke severity was also a strong predictor of adverse outcomes, including mortality, prolonged hospitalization, and stroke-related death, further supporting the prognostic validity of the NIHSS.

Nevertheless, the present findings showed no significant association between HbA1c categories and in-hospital mortality, prolonged hospital stay, or stroke-related deaths (*p* > 0.05). Our findings align with Lee et al., who reported no association between HbA1c (≥6.5%) and NIHSS severity, although their study also evaluated infarct volume, which was not assessed in the present analysis (26). Likewise, Shimoyama et al. demonstrated that elevated admission glucose, but not HbA1c, correlated with infarct progression and neurological deterioration (12). Taken together, these findings suggest that HbA1c may have limited utility in predicting acute neurological severity, despite its established role as a marker of chronic glycemic control and long-term vascular risk.

Conversely, several studies have reported associations between HbA1c and stroke severity. For example, Hjalmarsson et al. found a significant relationship using HbA1c > 6% and NIHSS ≥ 7 (13), while Abdalgbar and Altalhi observed similar findings in a smaller cohort (*n* = 80) with HbA1c > 7% (27). Variations in HbA1c thresholds, outcome measures, and patient populations likely explain these inconsistencies. Our study enhances methodological rigor by employing ADA-defined HbA1c categories, validated NIHSS cutoffs, and inclusion of both diabetic and non-diabetic patients. Moreover, studies assessing long-term outcomes with the modified Rankin Scale (mRS) consistently demonstrate that elevated HbA1c is linked to poorer recovery (11, 14). Collectively, this suggests that HbA1c may hold greater prognostic value for long-term outcomes rather than for acute stroke severity.

The absence of an association between HbA1c and acute stroke severity may reflect underlying pathophysiology. Diabetic complications are closely linked to microvascular complications such as CAD, retinopathy, nephropathy, and neuropathy (28, 29), whereas its direct impact on acute macrovascular events like stroke is less pronounced. Because HbA1c reflects average glycemia over the preceding 2–3 months, it may not capture the immediate metabolic and hemodynamic factors that influence infarct evolution at presentation. By contrast, admission hyperglycemia has been associated in prior studies with worse neurological outcomes, larger infarct volumes, and infarct progression, possibly through oxidative stress, endothelial dysfunction, and impaired cerebral autoregulation (30–34). Although acute glycemic markers were not evaluated in the present study, this distinction may help explain why chronic glycemic status, as reflected by HbA1c, was not associated with NIHSS-defined stroke severity in our cohort.

Overall, our findings support a more cautious interpretation of HbA1c in acute ischemic stroke. Rather than serving as an isolated predictor of neurological severity at presentation, HbA1c may be more informative as part of a broader metabolic and vascular risk profile. In contrast, established clinical tools such as the NIHSS remain more directly relevant for early assessment of stroke severity and in-hospital prognosis.

### 4.1 Limitations

This study has several limitations. Its retrospective design limits the ability to draw causal inferences. Functional outcomes beyond hospital discharge, such as the modified Rankin Scale (mRS), were not available, restricting comparisons with long-term prognostic studies. Neuroimaging parameters, including infarct volume and perfusion, were not assessed, which could have provided additional mechanistic insights. NIHSS scores at discharge were recorded for only 59.2% of patients, and the missing data may have introduced bias if these patients systematically differed from those with available scores. The study cohort also included a relatively high proportion of patients with known diabetes mellitus, which may have influenced the distribution of glycemic categories and limited comparisons with populations that include larger numbers of non-diabetic individuals. Finally, the relatively small proportion of patients with severe stroke may have reduced the statistical power of subgroup analyses.

### 4.2 Recommendations for future research

Future prospective, multicenter studies should evaluate both chronic glycemic markers, such as HbA1c, and acute markers, including glycated albumin and admission plasma glucose, to comprehensively assess the impact of glycemic status on stroke outcomes. The inclusion of neuroimaging parameters, such as infarct volume and penumbra viability, could provide deeper insight into underlying pathophysiological mechanisms. Additionally, long-term functional outcomes, particularly those measured by the modified Rankin Scale (mRS), should be systematically assessed to clarify the prognostic significance of HbA1c at different stages of stroke management.

## 5 Conclusion

In this large cohort, admission HbA1c levels were not associated with stroke severity. Although HbA1c remains important for long-term vascular risk assessment, it did not predict neurological status at presentation in this study Higher HbA1c categories were, however, associated with a greater burden of vascular comorbidities in unadjusted analysis, particularly hypertension, hyperlipidemia, coronary artery disease, and atrial fibrillation; however, these associations did not remain significant after multivariable adjustment. Consequently, established clinical assessment tools—particularly the NIHSS—remain the most reliable and practical instruments for early prognostic evaluation of stroke severity. These findings suggest that HbA1c may be better interpreted as a marker of chronic vascular and metabolic risk rather than as an independent predictor of acute stroke severity.

## Data Availability

The datasets generated and/or analyzed during the current study are not publicly available due to institutional and patient confidentiality restrictions but are available from the corresponding author on reasonable request.

## References

[1] Kannel WB McGee DL. Diabetes and cardiovascular disease. JAMA. (1979) 241:2035–8. doi: 10.1001/jama.241.19.2035 430798

[2] Stratton IM Adler AI Neil HAW Matthews DR Manley SE Cull CA . Association of glycaemia with macrovascular and microvascular complications of type 2 diabetes (UKPDS 35): prospective observational study. BMJ. (2000) 321:405–12. doi: 10.1136/bmj.321.7258.405, 10938048 PMC27454

[3] UK Prospective Diabetes Study (UKPDS) Group. Intensive blood-glucose control with sulphonylureas or insulin compared with conventional treatment and risk of complications in patients with type 2 diabetes (UKPDS 33). Lancet. (1998) 352:837–53. doi: 10.1016/S0140-6736(98)07019-6 9742976

[4] Mitsios JP Ekinci EI Mitsios GP Churilov L Thijs V. Relationship between glycated haemoglobin and stroke risk: a systematic review and meta-analysis. J Am Heart Assoc. (2018) 7:e007858. doi: 10.1161/JAHA.117.007858, 29773578 PMC6015363

[5] Vermeer SE Sandee W Algra A Koudstaal PJ Kappelle LJ Dippel DWJ . Impaired glucose tolerance increases stroke risk in nondiabetic patients with TIA or minor ischemic stroke. Stroke. (2006) 37:1413–7. doi: 10.1161/01.STR.0000221766.73692.0b 16627787

[6] Banerjee C Moon YP Paik MC Rundek T Mora-McLaughlin C Vieira JR . Duration of diabetes and risk of ischemic stroke: the northern Manhattan study. Stroke. (2012) 43:1212–7. doi: 10.1161/STROKEAHA.111.641381, 22382158 PMC3336044

[7] Bao Y Gu D. Glycated hemoglobin as a marker for predicting outcomes of patients with stroke (ischemic and hemorrhagic): a systematic review and meta-analysis. Front Neurol. (2021) 12:642899. doi: 10.3389/fneur.2021.642899, 33868148 PMC8044393

[8] Baird TA Parsons MW Barber PA Butcher KS Desmond PM Tress BM . The influence of diabetes mellitus and hyperglycaemia on stroke incidence and outcome. J Clin Neurosci. (2002) 9:618–26. doi: 10.1054/jocn.2002.1081, 12604269

[9] Koenig RJ Peterson CM Jones RL Saudek C Lehrman M Cerami A. Correlation of glucose regulation and hemoglobin A1c in diabetes mellitus. N Engl J Med. (1976) 295:417–20. doi: 10.1056/NEJM197608192950804, 934240

[10] Dong N Shen X Wu X Guo X Fang Q. Elevated glycated hemoglobin levels are associated with poor outcome in acute ischemic stroke. Front Aging Neurosci. (2022) 13:821336. doi: 10.3389/fnagi.2021.821336, 35185521 PMC8851318

[11] Lattanzi S Bartolini M Provinciali L Silvestrini M. Glycosylated hemoglobin and functional outcome after acute ischemic stroke. J Stroke Cerebrovasc Dis. (2016) 25:2189–94. doi: 10.1016/j.jstrokecerebrovasdis.2016.03.018, 27103269

[12] Shimoyama T Kimura K Uemura J Saji N Shibazaki K. Elevated glucose level adversely affects infarct volume growth and neurological deterioration in non-diabetic stroke patients, but not diabetic stroke patients. Eur J Neurol. (2014) 21:402–10. doi: 10.1111/ene.12280, 24517878

[13] Hjalmarsson C Manhem K Bokemark L Andersson B. The role of prestroke glycemic control on severity and outcome of acute ischemic stroke. Stroke Res Treat. (2014) 2014:694569. doi: 10.1155/2014/694569, 25295219 PMC4175748

[14] O’Donnell MJ Chin SL Rangarajan S Xavier D Liu L Zhang H . Global and regional effects of potentially modifiable risk factors associated with acute stroke in 32 countries (INTERSTROKE). Lancet. (2016) 388:761–75. doi: 10.1016/S0140-6736(16)30506-2 27431356

[15] American Diabetes Association. Standards of medical care in diabetes—2024. Diabetes Care. (2024) 47:S1–S225. doi: 10.2337/dc23-S00138078587

[16] Brott T Adams HP Jr Olinger CP Marler JR Barsan WG Biller J . Measurements of acute cerebral infarction: a clinical examination scale. Stroke. (1989) 20:864–70. doi: 10.1161/01.STR.20.7.864, 2749846

[17] Saber H Saver JL. Distributional validity and prognostic power of the National Institutes of Health stroke scale in US administrative claims data. JAMA Neurol. (2020) 77:606–12. doi: 10.1001/jamaneurol.2019.5061, 32065612 PMC7042858

[18] Adams HP Jr Davis PH Leira EC Chang KC Bendixen BH Clarke WR . Baseline NIH stroke scale score strongly predicts outcome after stroke. Stroke. (1999) 30:592–7. doi: 10.1212/WNL.53.1.126 10408548

[19] Grundy SM Stone NJ Bailey AL Beam C Birtcher KK Blumenthal RS . 2018 AHA/ACC guideline on the management of blood cholesterol: executive summary. J Am Coll Cardiol. (2019) 73:3168–93. doi: 10.1016/j.jacc.2018.11.002 30423391

[20] World Health Organization. Obesity: Preventing and Managing the Global Epidemic. Report of a WHO Consultation. WHO Technical Report Series 894. Geneva: World Health Organization (2000). p. 252. 11234459

[21] Cipolla MJ Liebeskind DS Chan SL. The importance of comorbidities in ischemic stroke: impact of hypertension on the cerebral circulation. J Cereb Blood Flow Metab. (2018) 38:2129–49. doi: 10.1177/0271678X18800589, 30198826 PMC6282213

[22] Chan YH Chuang C Chan CC Lee HF Huang YC Huang YT . Glycemic status and risks of thromboembolism and major bleeding in patients with atrial fibrillation. Cardiovasc Diabetol. (2020) 19:30. doi: 10.1186/s12933-020-01005-8, 32156277 PMC7063754

[23] Zhang A Deng W Zhang B Ren M Tian L Ge J . Association of lipid profiles with severity and outcome of acute ischemic stroke in patients with and without chronic kidney disease. Neurol Sci. (2021) 42:2371–8. doi: 10.1007/s10072-020-04791-x, 33048272 PMC8159792

[24] Tsagalis G Akrivos T Alevizaki M Manios E Stamatellopoulos K Laggouranis A . Renal dysfunction in acute stroke: an independent predictor of long-term combined vascular events and overall mortality. Nephrol Dial Transplant. (2009) 24:194–200. doi: 10.1093/ndt/gfn471, 18728156

[25] Wang H Chen S Li X Zhu Z Zhang W. Impact of elevated hemoglobin A1c levels on functional outcome in patients with acute ischemic stroke. J Stroke Cerebrovasc Dis. (2019) 28:470–6. doi: 10.1016/j.jstrokecerebrovasdis.2018.10.026, 30415918

[26] Lee SH Jang MU Kim Y Park SY Kim C Kim YJ . Effect of prestroke glycemic variability estimated by glycated albumin on stroke severity and infarct volume in diabetic patients with acute ischemic stroke. Front Endocrinol. (2020) 11:230. doi: 10.3389/fendo.2020.00230 PMC7186307 32373074

[27] Abdalgbar AA Altalhi HGK. Glycated hemoglobin level is significantly associated with the severity of ischemic stroke in Libyan non-diabetic and diabetic patients. ARC J Nutr Growth. (2019) 2:237–40. doi: 10.20431/2455-2550.0501003

[28] Aikaeli F Njim T Gissing S Moyo F Alam U Mfinanga SG . Prevalence of microvascular and macrovascular complications of diabetes in newly diagnosed type 2 diabetes in low- and middle-income countries: a systematic review and meta-analysis. PLOS Glob Public Health. (2022) 2:e0000599. doi: 10.1371/journal.pgph.0000599, 36962416 PMC10021817

[29] Kosiborod M Gomes MB Nicolucci A Chen H Cid-Ruzafa J Ivanova A . Incidence rates and predictors of microvascular and macrovascular complications in patients with type 2 diabetes: results from the global DISCOVER study. J Clin Transl Endocrinol. (2021) 26:100284. doi: 10.1016/j.ahj.2021.10.181

[30] Nalysnyk L Hernandez-Medina M Krishnarajah G. Glycaemic variability and complications in patients with diabetes mellitus: evidence from a systematic review. Diabetes Obes Metab. (2010) 12:288–98. doi: 10.1111/j.1463-1326.2009.01160.x, 20380649

[31] Ceriello A Esposito K Piconi L Ihnat MA Thorpe JE Testa R . Oscillating glucose is more deleterious to endothelial function and oxidative stress than mean glucose in normal and type 2 diabetic patients. Diabetes. (2008) 57:1349–54. doi: 10.2337/db08-0063, 18299315

[32] Quagliaro L Piconi L Assaloni R Martinelli L Motz E Ceriello A. Intermittent high glucose enhances apoptosis related to oxidative stress in human umbilical vein endothelial cells: role of PKC and NAD(P)H oxidase. Diabetes. (2003) 52:2795–804. doi: 10.2337/diabetes.52.11.2795, 14578299

[33] Piconi L Quagliaro L Assaloni R Da Ros R Maier A Zuodar G . Constant and intermittent high glucose enhances endothelial cell apoptosis through mitochondrial superoxide overproduction. Diabetes Metab Res Rev. (2006) 22:16453381:198–203. doi: 10.1002/dmrr.613 16453381

[34] Risso A Mercuri F Quagliaro L Damante G Ceriello A. Intermittent high glucose enhances apoptosis in human umbilical vein endothelial cells in culture. Am J Physiol Endocrinol Metab. (2001) 281:E924–30. doi: 10.1152/ajpendo.2001.281.5.E924, 11595647

[35] Megherbi SE Milan C Minier D Couvreur G Osseby GV Tilling K . Association between diabetes and stroke subtype on survival and functional outcome 3 months after stroke: BIOMED stroke project. Stroke. (2003) 34:688–94. doi: 10.1161/01.STR.0000057975.15221.40 12624292

